# Exploring the antibacterial potential of plant extracts and essential oils against *Bacillus thermophilus* in beet sugar for enhanced sucrose retention: a comparative assessment and implications

**DOI:** 10.3389/fmicb.2023.1219823

**Published:** 2023-07-20

**Authors:** Mohamed M. Yousef, Abdel-Naser A. Zohri, Amira M. G. Darwish, Abdelaal Shamseldin, Sanaa A. Kabeil, Ahmed Abdelkhalek, Reem Binsuwaidan, Mariusz Jaremko, Hussah Abdullah Alshwyeh, Elsayed E. Hafez, Essa M. Saied

**Affiliations:** ^1^Faculty of Sugar Industry Technology and Integrated Industries, Assiut University, Assiut, Egypt; ^2^Department of Botany and Microbiology, Faculty of Science, Assuit University, Assiut, Egypt; ^3^Food Industry Technology Program, Faculty of Industrial and Energy Technology, Borg Al Arab Technological University, Alexandria, Egypt; ^4^Food Technology Department, Arid Lands Cultivation Research Institute, City of Scientific Research and Technological Applications (SRTA-City), Alexandria, Egypt; ^5^Department of Environmental Biotechnology, GEBRI Institute at the City of Scientific Research and Technology Applications, New Borg El-Arab, Alexandria, Egypt; ^6^Department of Protein Research, GEBRI Institute at the City of Scientific Research and Technology Applications, Alexandria, Egypt; ^7^Plant Protection and Biomolecular Diagnosis Department, Arid Lands Cultivation Research Institute, City of Scientific Research and Technological Applications, New Borg El-Arab, Alexandria, Egypt; ^8^Department of Pharmaceutical Sciences, College of Pharmacy, Princess Nourah bint Abdulrahman University, Riyadh, Saudi Arabia; ^9^Division of Biological and Environmental Sciences and Engineering, Smart-Health Initiative and Red Sea Research Center, King Abdullah University of Science and Technology, Thuwal, Saudi Arabia; ^10^Department of Biology, College of Science, Imam Abdulrahman Bin Faisal University, Dammam, Saudi Arabia; ^11^Basic and Applied Scientific Research Center, Imam Abdulrahman Bin Faisal University, Dammam, Saudi Arabia; ^12^Chemistry Department, Faculty of Science, Suez Canal University, Ismailia, Egypt; ^13^Institute for Chemistry, Humboldt Universität zu Berlin, Berlin, Germany

**Keywords:** sugar beet, sucrose production, *Bacillus thermophilus*, medicinal plant extracts, essential oils, GC/MS analysis, DD-PCR

## Abstract

Sugar beet is one of the greatest sources for producing sugar worldwide. However, a group of bacteria grows on beets during the storage process, leading to a reduction in sucrose yield. Our study focused on identifying common bacterial species that grow on beets during manufacturing and contribute to sucrose loss. The ultimate goal was to find a potential antibacterial agent from various plant extracts and oils to inhibit the growth of these harmful bacteria and reduce sucrose losses. The screening of bacterial species that grow on beet revealed that a large group of mesophilic bacteria, such as *Bacillus subtilis, Leuconostoc mesenteroides, Pseudomonas fluorescens, Escherichia coli, Acinetobacter baumannii, Staphylococcus xylosus, Enterobacter amnigenus*, and *Aeromonas* species, in addition to a dominant thermophilic species called *Bacillus thermophilus*, were found to be present during the manufacturing of beets. The application of 20 plant extracts and 13 different oils indicated that the extracts of *Geranium gruinum, Datura stramonium*, and *Mentha spicata* were the best antibacterials to reduce the growth of *B*. *thermophilus* with inhibition zones equal to 40, 39, and 35 mm, respectively. In contrast, the best active oils for inhibiting the growth of *B*. *thermophilus* were *Mentha spicata* and *Ocimum bacilicum*, with an inhibitory effect of 50 and 45 mm, respectively. RAPD-PCR with different primers indicated that treating sugar juice with the most effective oils against bacteria resulted in new recombinant microorganisms, confirming their roles as strong antibacterial products. The characterization of *Mentha spicata* and *Ocimum bacilicum* oils using GC/MS analysis identified *cis*-iso pulegone and hexadecanoic acid as the two main bioactive compounds with potential antibacterial activity. An analysis of five genes using DD-PCR that have been affected due to antibacterial activity from the highly effective oil from *Mentha spicata* concluded that all belonged to the family of protein defense. Our findings indicate that the application of these pure antibacterial plant extracts and oils would minimize the reduction of sucrose during sugar production.

## 1. Introduction

Sugar beet (*Beta vulgaris* L.) is a highly versatile root crop grown primarily for sugar production (Subrahmanyeswari and Gantait, 2022). It is a biennial plant that belongs to the family *Amaranthaceae* and is related to other common crops such as chard and spinach. Sugar beet is a high-yielding crop grown in temperate regions worldwide, with major producers including Africa, Europe, the United States, and Russia (Kagami et al., 2015; Tayyab et al., 2023). The production of sugar beet typically involves a series of steps, from seed selection and planting to harvesting and processing (Tayyab et al., 2023). Sugar beet seeds are usually sown in the spring, with optimal planting times depending on the local climate and soil conditions. As plants grow, they require regular watering and fertilization to produce healthy roots (Modelska et al., 2020; Peighambardoust et al., 2021; Salelign and Duraisamy, 2021). Once sugar beet plants have matured, they are harvested and transported to processing facilities, where sugar is extracted. This extraction process involves a complex series of steps, including washing beets, followed by slicing and boiling the roots to extract the juice. The juice is then purified and concentrated, with the resulting sugar typically sold to manufacturers for use in various food products. In addition to its use as a source of sugar, sugar beet is also used in the production of animal feed and biofuel. The pulp and other byproducts of sugar beet processing can be used to produce feed for livestock, while the leftover sugar beet pulp can also be used as a feedstock for producing biofuels such as ethanol (Berłowska et al., 2016; Maravić et al., 2018; Mall et al., 2021; Usmani et al., 2022).

Sugar beet (*Beta vulgaris* L.) is a root crop that is widely grown for the production of sucrose, which is used in a variety of food and beverage products. However, significant amounts of recoverable sucrose can be lost during the long-term storage of beetroots or during the manufacturing process (Cole, 1977; Wyse et al., 1978; Akeson and Widner, 1981; McGinnis, 1982; Field, 1992; Bugbee, 1993; Harvey and Dutton, 1993; Van Eerd et al., 2012). This loss of sucrose can occur due to a number of different factors, including storage time, root quality, type of sugar beet cultivar used, respiration of roots, pre-harvest and harvest processes, and microbial infection (Lafta and Fugate, 2009; Fugate et al., 2016; Kusstatscher et al., 2019; Madritsch et al., 2020; Kleuker and Hoffmann, 2022). One of the primary factors that can lead to the loss of sucrose during storage is the length of time that beets are stored. As beets are stored for longer periods of time, the respiration rate of the roots increases, which can lead to the breakdown of sucrose and the loss of recoverable sugar (Campbell et al., 2008; Lafta and Fugate, 2009; Strausbaugh et al., 2009; Ruan et al., 2010; Aluko et al., 2021). Similarly, the quality of the roots can also play a significant role in the loss of sucrose during storage. Poor-quality roots, which may be damaged or diseased, can be more susceptible to microbial infection and other factors contributing to sucrose breakdown (Madritsch et al., 2020; Aluko et al., 2021). The type of sugar beet cultivar used can also impact the amount of sucrose that is lost during storage or the manufacturing process. Some cultivars are more susceptible to disease or other factors that can lead to the breakdown of sucrose, while others may have a higher inherent sugar content and thus be less likely to experience significant losses (El-Geddawy and El-Rahman, 2019; Barreto et al., 2021). Pre-harvest and harvest processes can also impact the amount of recoverable sucrose in sugar beets. For example, beets that are harvested too early may have a lower sugar content, while those that are harvested too late may be more susceptible to damage and microbial infection (Selmar and Kleinwächter, 2013). The method of harvesting, such as hand harvesting vs. mechanical harvesting, can also impact the quality of the beets and thus the amount of recoverable sugar. Finally, microbial infection can be a major contributor to the loss of sucrose in sugar beets. Microbes can consume the sugar in roots, leading to a reduction in the recoverable sucrose content. Proper sanitation and storage practices can help minimize the risk of microbial infection and reduce the loss of sucrose during storage and processing (Selmar and Kleinwächter, 2013; Kumar and Kalita, 2017; Misra et al., 2020, 2022).

The discovery of antimicrobial compounds present in plants has become an interesting topic in the scientific community due to their potential to provide natural alternatives to conventional synthetic antimicrobial agents. It has been reported that more than 1,340 plants have been identified to contain such compounds, but only a fraction of them have been extensively studied. The antimicrobial activity of plant extracts and oils has been investigated by several researchers to determine their effectiveness in inhibiting the growth of microorganisms such as bacteria and fungi (Wilkins and Board, 1989; Chao et al., 2000; Burt, 2004; Vági et al., 2004; El Azab et al., 2021; Helmy et al., 2023). The use of natural compounds as antimicrobial agents is of great interest due to the growing concern over the emergence of antibiotic-resistant microorganisms. In addition, the use of synthetic antimicrobial agents has been linked to negative effects on human health and the environment. Therefore, the search for natural sources of antimicrobial agents has gained significant attention (Refat et al., 2009; Gaber et al., 2021; Amaning Danquah et al., 2022; Qadri et al., 2022). Many studies have focused on identifying plant-derived compounds with antimicrobial properties and have demonstrated their potential to inhibit the growth of various microorganisms. These compounds have been extracted from different parts of plants, including leaves, roots, stems, and fruits. Additionally, some of these compounds have been found to possess additional beneficial properties, such as anti-inflammatory and antioxidant activities (Cowan, 1999; Barbieri et al., 2016; Vaou et al., 2021).

For example, tea tree oil *(Melaleuca alternifolia*) has been shown to have strong antimicrobial properties against bacteria such as *Staphylococcus aureus* and *Escherichia coli*, as well as fungi such as *Candida albicans*. This oil is rich in terpenes, including terpinen-4-ol, which has been identified as a major component responsible for its antimicrobial properties (Cox et al., 2000, 2001; Carson et al., 2006). Another example is garlic (*Allium sativum*), which contains allicin, a sulfur-containing compound that has broad-spectrum antimicrobial properties against bacteria, fungi, and viruses (Ankri and Mirelman, 1999; El-Saber Batiha et al., 2020; Bhatwalkar et al., 2021; Magryś et al., 2021). In addition to these natural compounds, plant extracts have also been shown to have antimicrobial activity. For instance, extracts from grapefruit seeds (*Citrus paradisi*) contain flavonoids, limonoids, and other compounds that have been found to inhibit the growth of bacteria such as *Staphylococcus aureus* and *Pseudomonas aeruginosa*, as well as fungi such as *Candida albicans*.

Similarly, extracts from oregano (*Origanum vulgare*) contain phenolic compounds such as carvacrol and thymol, which have been shown to have strong antimicrobial properties against various microorganisms, including bacteria, fungi, and viruses. Singh et al. (1994) showed that *Mentha spicata* plant extracts possess the ability to reduce the growth of *Fusarium oxysporum*, while an extract of *Mentha arvensis* proved its ability to limit the growth of *Fusarium pallidorosum* (Mohamed et al., 1996). Similarly, El-Korashy et al. reported that the extract of *Mentha spicata* inhibited the growth of *Rhizoctonia solani, Fusarium solani*, and *Sclerotium rolfsii* (El-Korashy, 1997).

Plant-derived antimicrobial compounds are not limited to medical and pharmaceutical applications. These compounds have also been studied for their potential in food preservation as natural alternatives to synthetic preservatives. For example, essential oils such as oregano and thyme have been found to have antimicrobial properties against foodborne pathogens such as *Salmonella* and *Listeria* monocytogenes and may be used to extend the shelf life of perishable food products (Jayasena and Jo, 2013; Mith et al., 2014; Yousefi et al., 2020; Angane et al., 2022).

Our interests are directed at microbial infections that cause a great loss in the sugar production of beet. Several chemical reagents and compounds, such as formalin, glutaraldehyde, and carbamates, have been used to limit microbial infection loads and reduce sugar loss. However, they have a notorious effect on human health. Therefore, finding a natural source that can substitute for chemical use is necessary, and it should be safe and applicable (Misra et al., 2020). Therefore, our goals were to explore the most common microorganisms that could attack sugar fruits and cause the loss of sugar during storage or production. Further, we aimed to discover novel natural antibacterial products from plant extracts or oils that can be used to reduce the detrimental effects of microbial growth. Lately, we have aimed to explore the mode of action for the antibacterial activity by applying molecular tools, including RAPD and DD-PCR analysis, to identify and confirm genes that the plant extracts or oils could target.

## 2. Results

### 2.1. Isolation and identification of bacteria attacking sugar beet during storage or manufacturing

A total of 32 different isolates of mesophilic bacteria were isolated and selected from the locations of companies as eight representative isolates for each location (Supplementary Table S1). These isolates were biochemically analyzed. Microscopically, the examination of fresh cultures of these bacterial isolates divided them into two major groups: bacilli (21 isolates) and cocci (11 isolates). Moreover, 17 of them were Gram-positive, and the rest were Gram-negative.

All collected bacterial isolates (32 isolates) were identified using MicroScan Panels (Table 1). The results of MicroScan Panels confirmed that all strains were negative for H2S production, and the remaining tests showed variable results among bacterial isolates. The results of MicroScan revealed that six isolates were identified as *Bacillus subtilis*, seven as *Leuconostoc mesenteroides*, four as *Pseudomonas fluorescens*, four as *Escherichia coli*, two as *Acinetobacter baumannii*, four as *Staphylococcus xylosus*, and three as *Enterobacter amnigenus*, in addition to one unidentified species called *Aeromonas* with two isolates. *Acinetobacter baumannii* was present in Dakahlia and Kafr El-Sheikh regions, as were *Aeromonas* species isolated from samples collected from Sharkia and Alexandria regions, while *Staphylococcus xylosus, L. mesenteroides, P. fluorescens*, and *B. subtilis* were common in the four study regions. *Escherichia coli* were found in samples from Dakahlia, Alexandria, and Kafr El-Sheikh regions, but *Enterobacter amnigenus* was isolated in Dakahlia, Sharkia, and Kafr El-Sheikh regions.

**Table 1:** A brief biochemical description of the eight identified bacterial species based on the MicroScanpanele results.

**Bacterial species**	**Biochemical results**																								
	**Gram stain**	**Catalase secretion**	**Oxidase secretion**	**Glucose fermentation**	**Xylose fermentation**	**Mannitol fermentation**	**Sucrose fermentation**	**Galactose fermentation**	**Mannose fermentation**	**Lactose fermentation**	**Maltose fermentation**	**Raffinose fermentation**	**L – Arabinose fermentation**	**Inulin fermentation**	**Indole**	**H**_2_ **S production**	**ONPG**	**Urea utilization**	**Nitrate reduction**	**Citrate utilization**	**Methyl red test**	**V.P test**	**Starch hydrolysis**	**Casein hydrolysis**	**Gelatin liquefaction**
*Bacillus subtilis*	+	+	+	+	−	+	+	−	−	−	+	+	+	+	−	−	−	−	+	+	−	+	+	+	+
*Leuconostoc mesenteroides*	+	−	−	+	+	−	+	+	−	+	+	−	−	−	−	−	−	−	−	+	−	−	−	−	−
*Pseudomonas fluorescens*	−	+	+	+	+	−	−	+	+	−	−	−	+	−	−	−	−	−	+	+	+	−	−	+	+
*Escherichia coli*	−	+	−	+	−	+	−	−	−	+	−	−	+	−	−	−	+		+	−	+	−	−	−	−
*Aeromonas species*	−	+	+	+	+	+	+	+	−	−	+	−	−	−	+	−	−	−	+	+	−	+	+	−	+
*Acinetobacter baumannii*	−	+	−	+	+	−	−	+	+	+	−	−	−	−	−	−	−	−	−	+	−	−	−	−	−
*Staphylococcus xylosus*	+	+	−	+	−	+	+	−	+	−	+	−	−	−	−	−	−	+	+	−	−	+	−	−	−
*Enterobacter amnigenus*	−	−	−	−	+	−	+	−	−	+	−	−	−	−	−	−	−	−	−	+	−	+	−	−	−

Brief biochemical descriptions of the eight identified bacterial species based on the MicroScan panels' results are shown in Table 1. Ten thermophilic isolates were isolated from beetroot samples collected from four companies after growing on nutrient agar at temperatures ranging from 55°C to 70°C. All of them were bacilli in shape and were Gram-positive and spore-forming. The biochemical and physiological characteristics of these isolates indicated that all of them could grow on casein and could not grow in Voges–Proskauer broth medium (Table 2). Moreover, they could reduce nitrate and convert hydrogen peroxide. All isolates could hydrolyze starch except isolate 2.

**Table 2:** Characteristics of the thermophilic bacterial isolate (*Bacillus thermophilus*) recovered from sugar beet roots during storage or manufacturing.

**Isolates no. tests**	**1**	**2**	**3**	**4**	**5**	**6**	**7**	**8**	**9**	**10**
**Morphology**	**All Bacilli shape**									
Gram stain	+	+	+	+	+	+	+	+	+	+
Catalase	+	+	+	+	+	+	+	+	+	+
Anaerobes	−	−	−	−	−	−	−	−	−	−
Spore forming	+	+	+	+	+	+	+	+	+	+
Starch hydrolysis	+	−	+	+	+	+	+	+	+	+
Casein	+	+	+	+	+	+	+	+	+	+
Gelatin	+	+	+	+	+	+	+	+	+	+
Voges-Proskauer	−	−	−	−	−	−	−	−	−	−
Nitrate reduced to nitrite	+	+	+	+	+	+	+	+	+	+
30°C	−	−	−	−	−	−	−	−	−	−
50°C	+	+	+	+	+	+	+	+	+	+
55°C	+	+	+	+	+	+	+	+	+	+
65°C	+	+	+	+	+	+	+	+	+	+
70°C	+	+	+	+	+	+	+	+	+	+
75°C	−	−	−	−	−	−	−	−	−	−

### 2.2. Molecular identification of thermophilic bacteria using 16S rRNA

**Figure 1:**
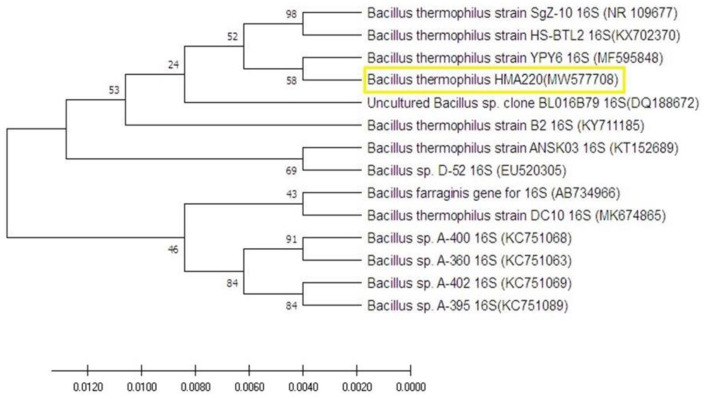
The molecular phylogenetic tree on the nucleotide sequence of *Bacillus thermophilus* HMA220 (MW577708) was obtained from a gene bank in yellow color. The phylogenetic tree was tested using bootstrap 2000 replications based on the UPGMA statistical method.

An amplified fragment of 350 bp of the 16S rRNA gene was amplified from the selected thermophilc bacterial isolate as a representative one, which was previously identified as *Bacillus themophilus* using biochemical analysis. This fragment was sequenced using the forward primer previously used for amplification, and the generated sequences were submitted to BLASTN to find homology with other bacterial 16S rRNA sequences from the Gene Bank. The homology of sequence analysis identified this strain as *Bacillus themophilus* HMA 220 with an accession number (MW577708). The phylogenetic tree with the closest species based on the UPGMA method is shown in Figure 1 using MEGA 10.

### 2.3. Assessment of the antibacterial activity of the plant extracts and oils against the growth of B. thermophilus

**Figure 2:**
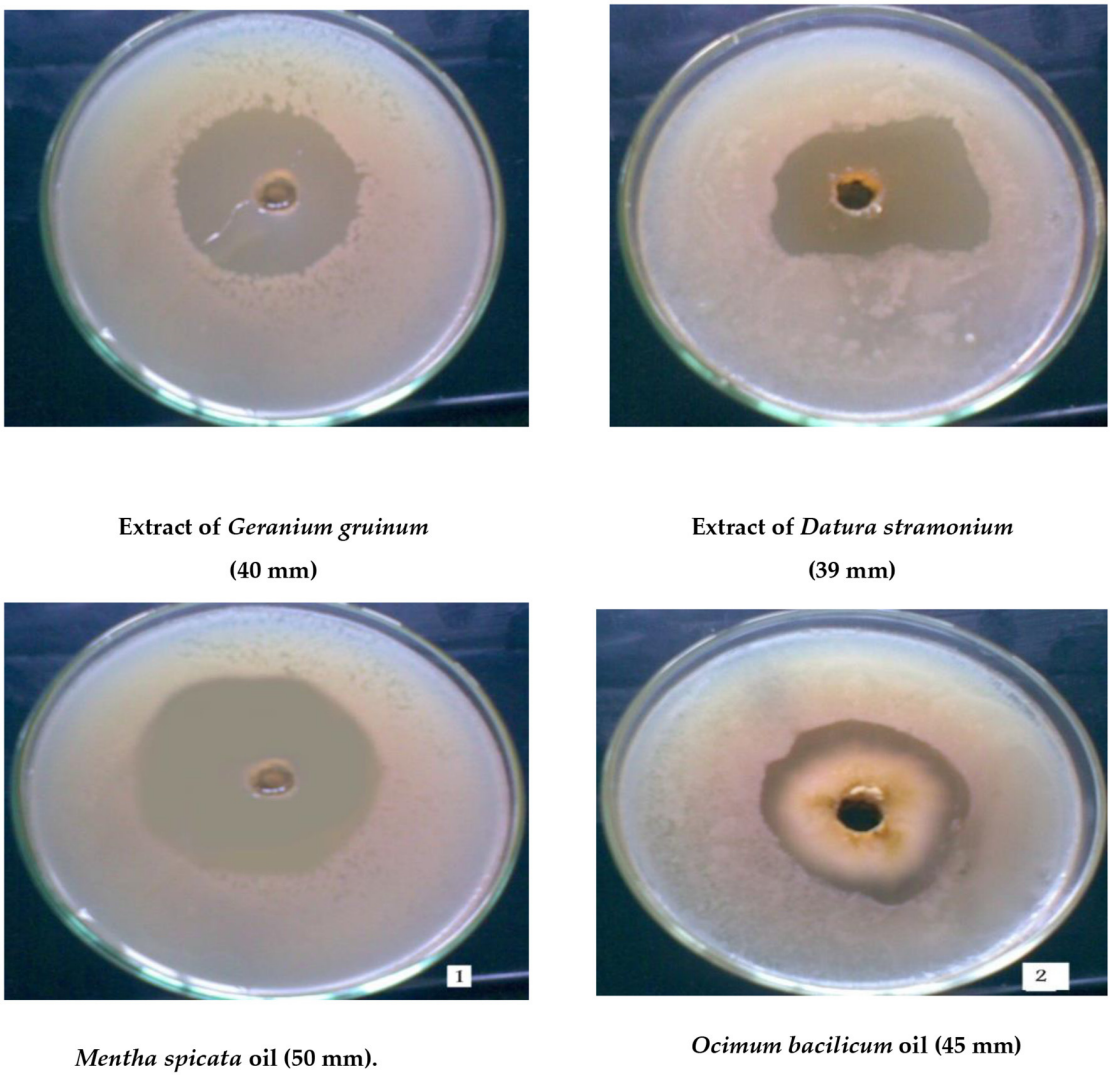
Maximum inhibition zone of cells of the thermotolerant strain (*Bacillus thermophilus*) as a result of treatment with the two highly effective plant extracts or oils.

A total of 20 different plant extracts were examined as antibacterial substances to inhibit the growth of *Bacillus thermophilus* because it was a major microorganism causing sugar losses. The results revealed that 18 plant extracts effectively inhibited the growth of *Bacillus thermophilus* (Table 3), and this was shown by forming an inhibition zone around the growth of the microbe; however, two plant extracts (*Mentha longifolia* L. and *Nerium oleander* L.) were not effective at all. Data on plant extracts showed that the most effective plant extracts against the growth of *Bacillus thermophilus* were recorded with extracts of *Geranium gruinum* (40 mm), followed by *Datura stramonium* (39 mm), and *Mentha spicata* (35 mm) (Figure 2 and Table 3). On the contrary, the lowest inhibition effect was observed with the extract from *Cymbopogon proximus* (hochst) staps (15 mm) (Table 3). The rest of the other plant extracts exhibited moderate inhibition of the growth of the thermal bacterial strain (*B. thermophilus*), and their inhibition zones ranged from 30 to 34 mm (Table 3).

**Table 3:** Effect of different plant extracts or oils on the growth of *Bacillus thermophilus*.

**No**.	**The plant extracts**	**Diameter of inhibition zone (mm)**	**Oils**	**Diameter of inhibition zone (mm)**
Control	DMSO (negative control)	ND	DMSO (negative control)	ND
1	*Ocimum basilicum* L.	33	*Mentha spicata* L.	50
2	*Citrus limon* L.	34	*Ocimum bacilicum*	45
3	*Cinnamomum zeylanicum* L.	22	*Cinnamomum zeylanicum* Blume	25
4	*Cuminum cyminum* L.	25	*Citrus limon*	23
5	*Eugenia caryophyllus* L.	33	*Origanum majorana*	22
6	*Mentha spicata* L.	35	*Eugenia caryophyllus*	20
7	*Eucalyptus rostrata* Schlecht.	33	*Eucalyptus rostrata* Schlecht.	20
8	*Geranium gruinum* L.	40	*Pimpinella schweinfurthii* Asch.	19
9	*Lantana camara* L.	25	*Olea europaea*	18
10	*Cymbopogon proximus* (Hochst) staps.	15	*Thymus vulgaris* L.	14
11	*Datura stramonium* L.	39	*Allium sativum*	13
12	*Nicotiana glauca* R.C. Graham	23	*Apium graveolens* L.	ND
13	*Silybum marianum* L. Gaertn	30	*Prunus dulcis* L.	ND
14	*Mentha longifolia* L.	ND		
15	*Humulus lupulus* L.	33		
16	*Schinus terebenthifolius* Radd	26		
17	*Nerium oleander* L.	ND		
18	*Zingiber officinale* L.	33		
19	*Caltrops procera* L.	23		
20	*Calendula officinalis* L.	25		

A total of 13 different oils were screened for their ability to inhibit the growth of *B. thermophilus* (Table 3). The results indicated that oils of *Mentha spicata* showed the highest inhibitory effect against *B. thermophilus* with an inhibition zone of 50 mm, followed by oils of *Ocimum bacilicum* with an inhibition zone of 45 mm (Figure 2). The other five oils (*Cinnamomum zeylanicum, Origanum majorana, Citrus limon, Eugenia caryophyllus*, and *Eucalyptus rostrata*) had a moderate effect on the tested strain with inhibition zones of 25, 23, 22, 20, and 20 mm, respectively, while oils from *Pimpeniella anisum, Olea europaea, Thymus vulgaris*, and *Allium sativum* were very less effective against *B. thermophilus* with inhibition zones of 13–19 mm (Table 3). The remaining two oils, *Apium graveolens* and *Prunus dulcisvar*, had no effect on the tested organism (Table 3).

Based on our findings, the most active antibacterial plant extracts are *Geranium gruinum* and oils from *Mentha spicata* and *Ocimum basilicum* against the growth of *B. thermophilus*. Subsequently, it was necessary to test whether the addition of these inhibitory products would affect the properties of the juice. Interestingly, it was noted that the pH of the juice increased (up to pH 5.75) compared to the control (pH 4.2), which was accompanied by an increase in the amount of sucrose (18.4%) compared to the control (15.2%). On the contrary, treatment with either *Ocimum basilicum* oil or the plant extract of *Geranium gruinum* showed no significant effect on the pH value (pH 4.2) and, subsequently, did not induce any considerable increase in the percentage of sucrose compared to the control.

### 2.4. Evaluation of lactic acid concentration in diffusion juice

The decrease in lactic acid concentration due to the growth of thermophilic bacteria was considered to be an indication of sucrose losses because of degradation. The highest ranges of lactic acid concentration (660 mg.L^−1^) were noted after 72 h of incubation. However, adding oils from *Mentha spicata, Ocimum bacilicum*, and *Cinnamomum zeylanicum* reduced lactic acid by 47.7%, 26.5%, and 35.6%, respectively.

### 2.5. GC/MS analysis of *Mentha spicata* and *Ocimum basilicum*

**Figure 3:**
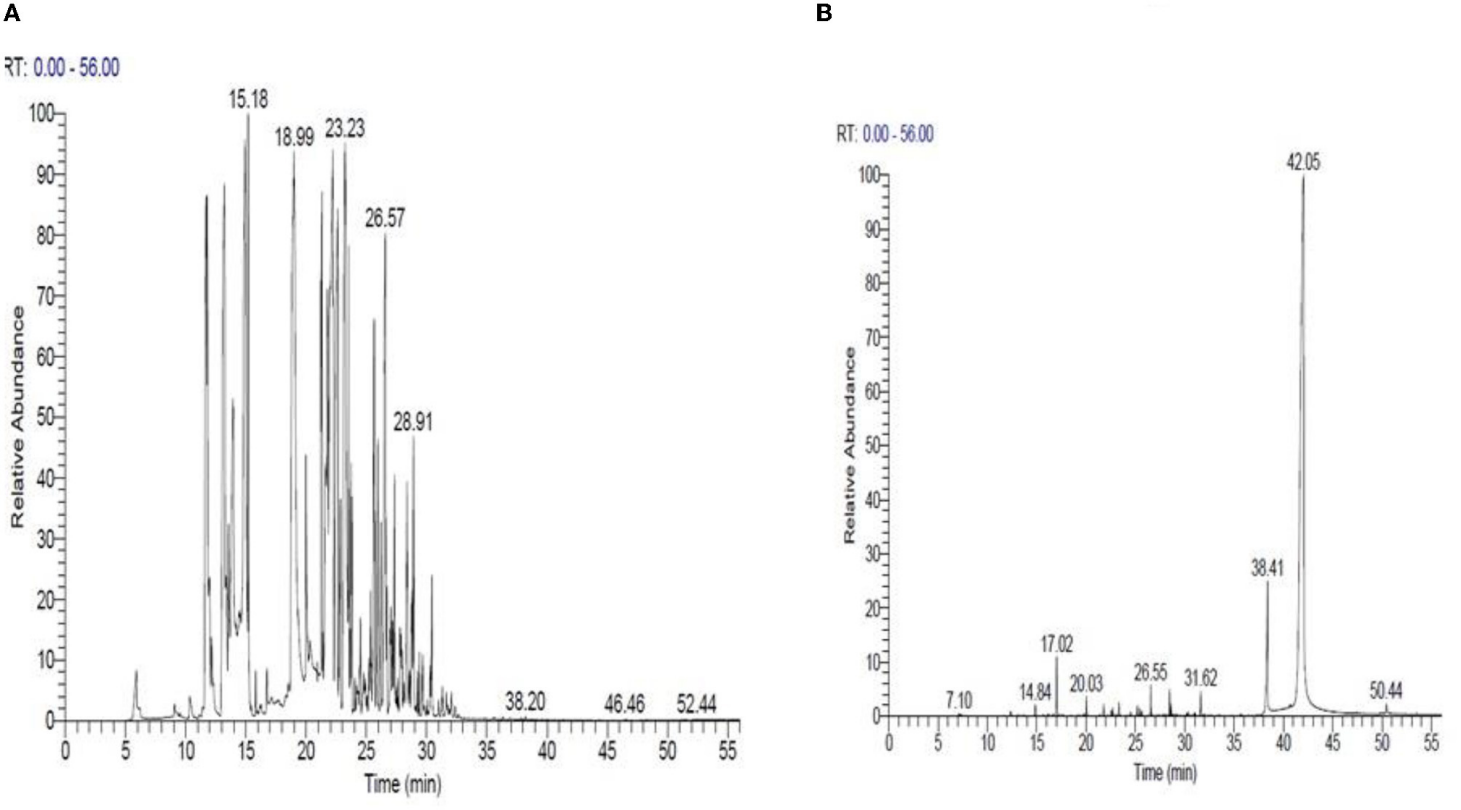
GC/MS of *Mentha spicata* essential oils **(A)** and *Ocimum basilicum* essential oils **(B)**, respectively.

Based on these findings, *Mentha spicata* and *Ocimum basilicum* oils were selected to further investigate their phytochemical characteristics. To obtain insights into the bioactive contents of the most active oils, we conducted GC/MS analyses for *Mentha spicata* oil and *Ocimum basilicum* oils. The results of GC/MS analysis indicated that 28 compounds were recognized in *Mentha spicata* oil Figure 3A. The most abundant components found in the *Mentha spicata* oil were *cis*-isopulegone (12.29%), followed by 3methyl-6-(1-methylethyl)-2-cyclohexen-1- one (8.41%), α-myrcene (6.69%), limonene (6.22%), methyl acetate (6.15%), γ-terpinene (5.43%), α-farnesene (4.49%), 5-methyl-2-isopropyl-2-hexenal (4.29%), and 5-methyl-3-heptanol (3.44%) (Table 4). On the other hand, 23 constituents were detected in *Ocimum basilicum*, representing 98.02% of the oil (Figure 3B). Cis-13-octadecenoic acid (81.53%), followed by hexadecanoic acid (6.66%) and L-linalool (2.22%), were found as major compounds. The whole chemical composition of the oil of *Ocimum basilicum*, as analyzed by GC and MS, is listed in Table 5.

**Table 4:** GC/MS data of the components of the essential oils of *Mentha spicata*.

**Peak No**.	**Component Name**.	**RT**	**Area %**	**M.F**.
1	γ-Terpinene	11.68	5.43	C_10_H_16_
2	Delta-3-Carene	11.78	2.47	C_10_H_16_
3	Cyclofenchene (CAS)	11.97	0.44	C_10_H_16_
4	Z-6-nonenal	12.15	0.35	C_9_H_16_O
5	α-Myrcene	13.21	6.69	C_10_H_16_
6	Linalyl acetate	13.37	0.38	C_12_H_20_O_2_
7	α-Myrcene	13.55	0.90	C_10_H_16_
8	5methyl - 3-Heptanol	13.92	3.44	C_8_H_18_O
9	Limonene	14.93	6.22	C_10_H_16_
10	1,8-Cineole	15.19	1.41	C_10_H_18_O
11	5-methyl-2-isopr opyl-2-hexenal	18.98	4.29	C_10_H_18_O
12	2(5-Hexenyl) cyclopentanone	19.98	1.05	C_11_H_18_O
13	Hexyl alcohol	21.29	4.12	C_6_H_14_O
14	Carveol 1	21.76	2.74	C_10_H_16_O
15	Cis-isopulegone	22.21	12.29	C_10_H_16_O
16	3-methyl-6-(1-methylethyl)-2-cyclohexen-1-one	22.62	8.41	C_10_H_16_O
17	Menthyl acetate	23.22	6.15	C_12_H_22_O_2_
18	Isomenthyl acetate	23.73	1.01	C_12_H_22_O_2_
19	Isopulegol acetate	23.79	0.75	C_12_H_20_O_2_
20	3-(4-methyl-3-pentenyl)-Furan	24.51	0.66	C_10_H_14_O
21	α- Bourbonene	25.64	3.40	C_15_H_24_
22	Longifolene	25.94	1.48	C_8_H_16_O_2_
23	α-Farnesene	26.56	4.49	C_15_H_24_
24	Longiborn-9- ene	26.96	0.37	C_15_H_24_
25	Sabinene	27.33	1.46	C_10_H_16_
26	γ-Muurolene	28.37	1.86	C_15_H_24_
27	ë-Cadinene (CAS)	28.91	1.72	C_15_H_24_
28	Caryophyllene oxide	30.44	0.84	C_15_H_24_O
Total			85.98	

RT, retention time; M.F., molecular formula.

**Table 5:** GC/MS data of the components of the essential oils of *Ocimum basilicum*.

**Peak No**.	**Component Name**.	**RT**	**Area %**	**M.F**.
1	2,4-dimethyl hexane	7.09	0.20	C_8_H_18_
2	(Z)-4-Heptenal	12.33	0.25	C_7_H_12_O
3	1,8-Cineole	14.84	0.52	C_10_H_18_O
4	Linalool oxide	16.17	0.10	C_10_H_18_O_2_
5	L-Linalool	17.02	2.22	C_10_H_18_O
6	α-Terpinenyl acetate	19.85	0.06	C_12_H_20_O_2_
7	Estragole	20.03	0.72	C_10_H1_2O_
8	(Z)-2-Decenal	21.78	0.41	C_10_H_18_O
9	Bornyl acetate	22.55	0.19	C_12_H_20_O_2_
10	(E, E)-2,4-Decadienal	22.69	0.29	C_10_H_16_O
11	(E, Z)-2,4-Decadienal	23.32	0.55	C_10_H_16_O
12	α-Elemene	25.46	0.25	C_15_H_24_
13	(Methyleugenol)	25.65	0.19	C_11_H_14_O_2_
14	(Z, E)-α-farnesene	26.55	0.96	C_15_H_24_
15	1,2,3,4,4a,5,6,8-aoct ahydro-7-methyl Naphthalene 4-methylene-1-(1-methylethyl)-, (1à,4aá,8aà)	28.61	0.41	C_15_H_24_
16	2,3-Dihydro3methyl-2-(2propenyl)-4-H1b enzopyran4one	28.81	0.11	C_13_H_14_O_2_
17	8-Cedren-13-ol	30.20	0.10	C_15_H_24_O
18	Caryophyllene oxide	30.35	0.15	C_15_H_24_O
19	Cubenol	31.04	0.10	C_15_H_26_O
20	Tau.Cadinol	31.62	0.85	C_15_H_26_O
21	Junipene	31.94	0.10	C_15_H_24_
22	Hexadecanoic acid	38.40	6.66	C_16_H_32_O_2_
23	Cis-13-Octadecenoic acid	42.04	81.53	C_18_H_34_O_2_

### 2.6. Assessment of gene expression of *B. thermophilus* by RAPD-PCR after treatment with oils

**Figure 4:**
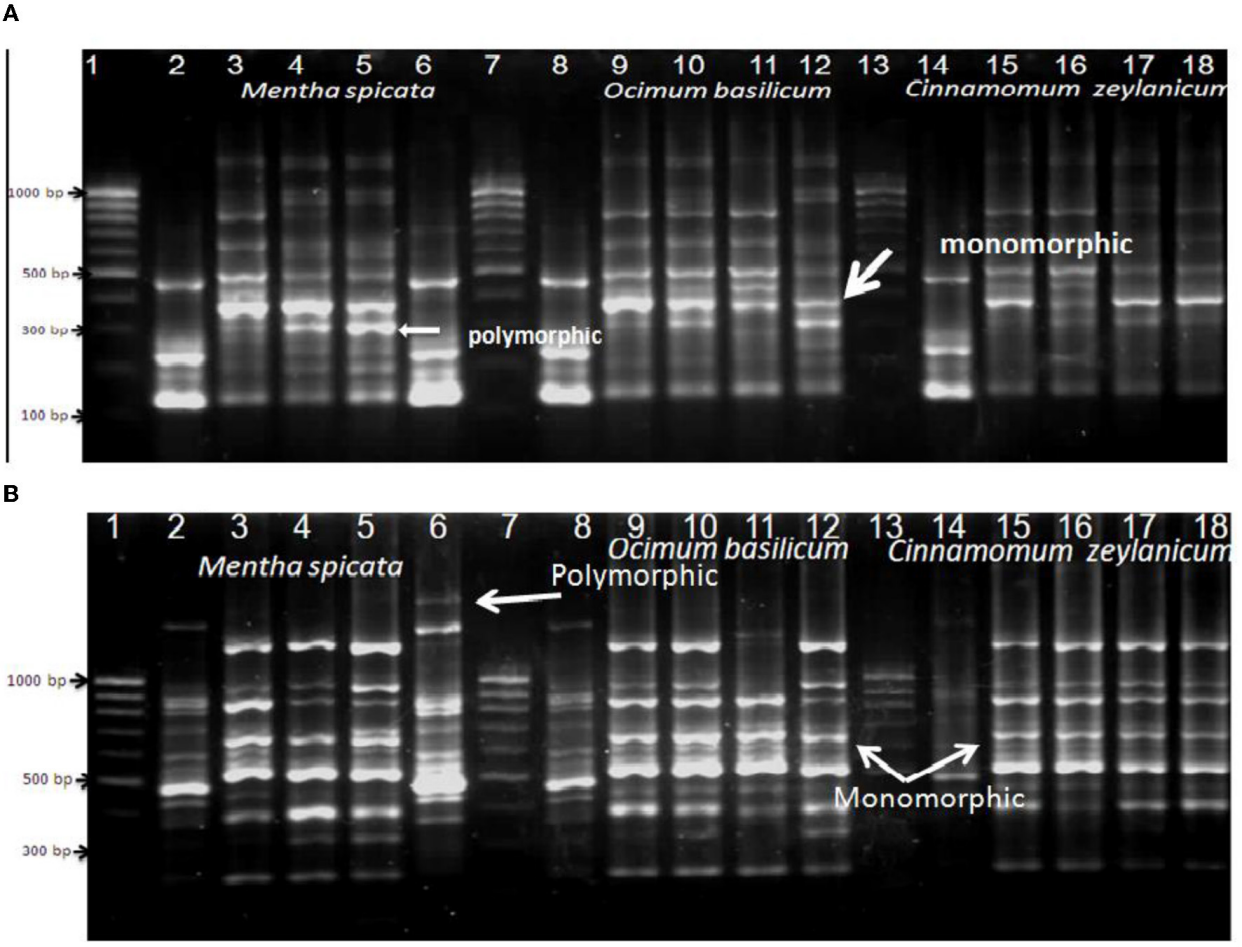
RAPD-PCR of bacterial cells of *Bacillus thermophilus* treated and non-treated with oils using primers A1 **(A)** and Ez351 **(B)**; Lanes 1, 7, and 13 are DNA markers of a 100-bp ladder, Lanes 2, 8, and 14: control (non-treated bacterial cells), Lanes 3, 4, 5, and 6 were treated with different concentrations of *Mentha spicata*, Lanes 9, 10, 11, and 12 were treated with different concentrations of *Ocimum basilicum*, and Lanes 15, 16, 17, and 18 were treated with different concentrations of *Cinnamomum zeylanicum*oil. Lanes 3, 9, and 15 were treated with 1/25 oil, Lanes 4, 10, and 16 with 1/50 oil, and Lanes 5, 11, and 17 with 1/100 oil.

Random amplified polymorphic DNA-polymers chain reactions (RAPD-PCR) were performed to know the genetic changes that can be present for the thermophilic strain (*B. thermophilus*) after treating it with different concentrations of *Mentha spicata, Ocimum basilicum*, and *Cinnamomum zeylanicum* oils in beet juice (1/25, 1/50, 1/100, and 1/200). The data presented in Figure 4A show that primer A1 provided 15 different band patterns with *Mentha spicata* oil treatment. Among these, 12 patterns showed polymorphic bands, while three bands were monomorphic. When treated with *Ocimum basilicum* oil, primer A1 generated 13 band patterns, all of which exhibited polymorphic bands except for two monomorphic bands.

Bacterial cells treated with the oil of *Cinnamomum zeylanicum* produced seven polymorphic bands and three monomorphic bands. The data presented in Figure 4B show that when primer EZ351 was used with the microbe and treated with *Mentha spicata* oil, it resulted in 18 band patterns. Out of these patterns, 11 displayed polymorphic bands, indicating genetic variation, while seven patterns exhibited monomorphic bands that remained consistent across all samples. Similarly, treating the thermal microorganism with *Ocimum basilicum* oil produced eight polymorphic and seven monomorphic bands. Furthermore, the application of *Cinnamomum zeylanicum* yielded five polymorphic and seven monomorphic bands.

**Figure 5:**
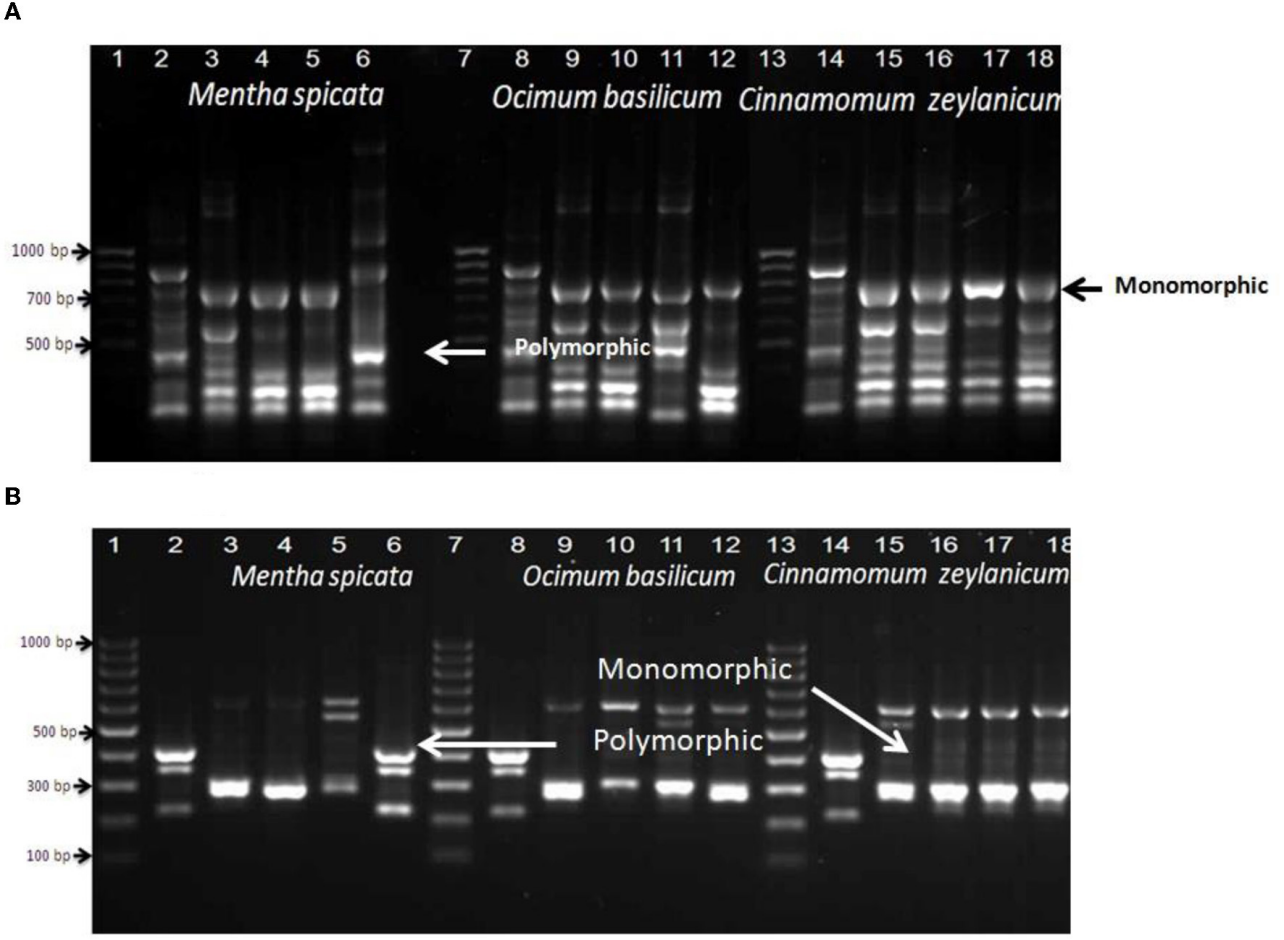
RAPD-PCR of bacterial cells of *Bacillus thermophilus* treated and non-treated with oils using primers Rapid2 **(A)** and A7A10 **(B)**; Lanes 1, 7, and 13 are DNA markers of a 100-bp ladder, Lanes 2, 8, and 14: control (non-treated bacterial cells), Lanes 3, 4, 5, and 6 were treated with different concentrations of *Mentha spicata*, Lanes 9, 10, 11, and 12 were treated with different concentrations of *Ocimum basilicum*, and Lanes 15, 16, 17, and 18 were treated with different concentrations of *Cinnamomum zeylanicum*oil. Lanes 3, 9, and 15 were treated with 1/25 oil, Lanes 4, 10, and 16 with 1/50 oil, and Lanes 5, 11, and 17 with 1/100 oil.

The data presented in Figure 5A show that primer RAPD 2 induced nine monomorphic and four polymorphic bands after treating *B. thearothermophilus* with *Mentha spicata* oil while using the oil of *Ocimum basilicum* as an antibacterial against *B. thearothermophilus* produced seven monomorphic and three polymorphic bands. The addition of *Cinnamomum zeylanicum* oil to inhibit the growth of the thermophilic *Bacillus* strain produced seven monomorphic and three polymorphic bands. The data presented in Figure 5B show that primer A7A10 succeeded in matching six polymorphic bands when the tested strain was treated with oil of *Mentha spicata*. In the case of using *Ocimum basilicum* oil, the primer produced seven polymorphic and only one monomorphic band.

### 2.7. Assessment of gene expression of *B. thermophilus* by differential display-PCR after treatment with *Mentha spicata* oil

**Figure 6:**
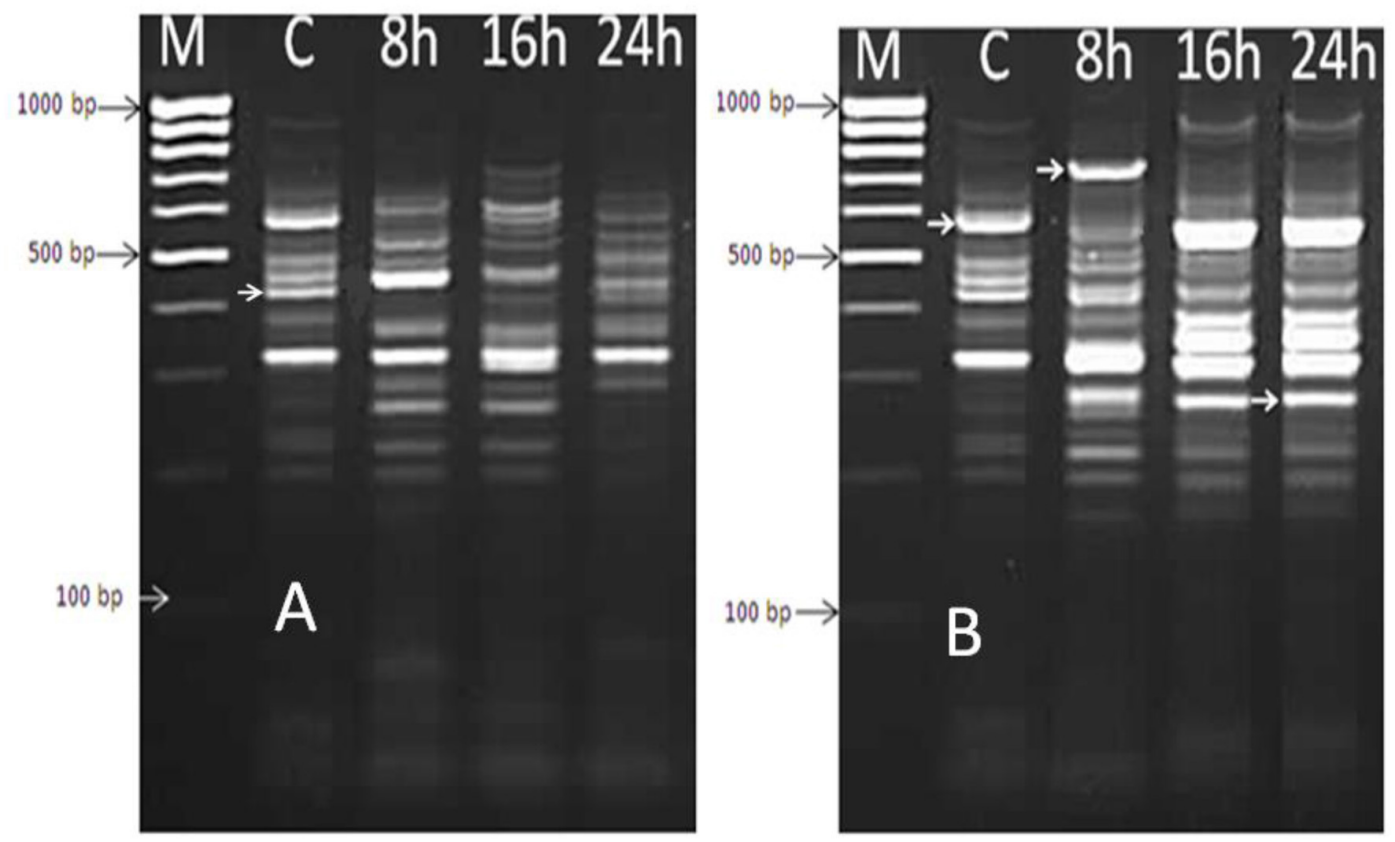
DD-PCR for the treated and non-treated bacterial cells of *Bacillus thermophilus* with oil using primers RAPD1 **(A)** and RAPD2 primer **(B)**; Lane 1 (M): molecular weight markers of a 100-bp ladder, Lane 2 (C): control cells, Lanes 3,4, and 5 refer to cells treated with *Mentha spicata* oil after 8 h, 16 h, and 24 h after the start of the incubation period.

**Figure 7:**
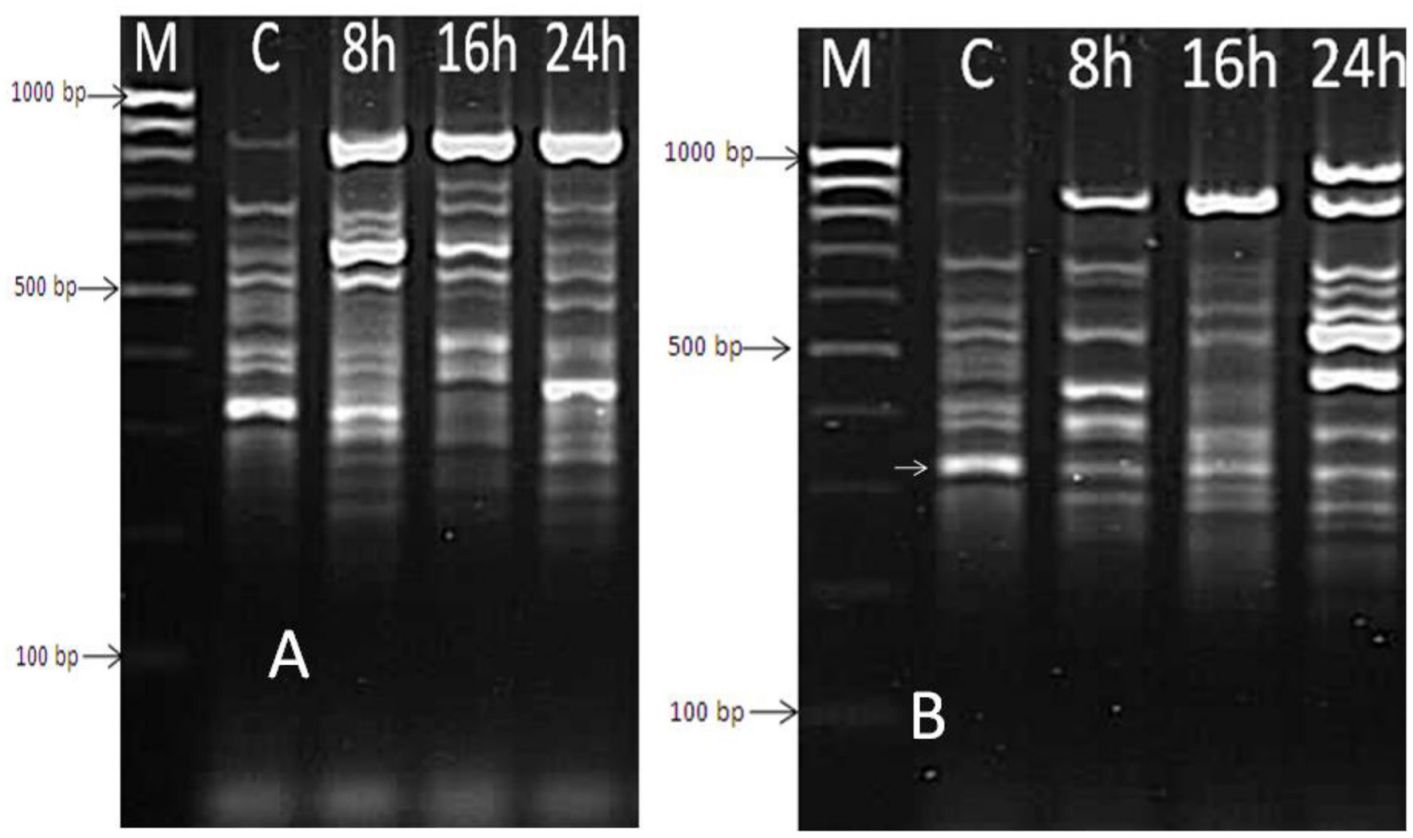
DD-PCR for the treated and non-treated bacterial cells of *Bacillus thermophilus* with oil using primers RAPD3 **(A)** and RAPD4 **(B)**; Lane 1 (M): molecular weight markers of a 100-bp ladder, Lane 2 (C): control cells; Lanes 3, 4, and 5 refer to the cells treated with *Mentha spicata* oil after 8 h, 16 h, and 24 h from incubation time.

Extracted mRNA from the bacterial cells of a selected isolate (*B. thermophilus*) treated with the effective oil of *Mentha spicata* was used to synthesize cDNA using primers RAPD1, RAPD2, RAPD3, and RAPD4 (DD-PCR). Variation in gene occurrence and density of the selected strain due to treatment with antibacterial products was carefully examined after the amplification of cDNA by comparing bands in a gel at different times of treatment (0, 8, 16, and 24 h). A number of such unique bands that were only present in treated samples and absent in non-treated samples were observed. Data presented in Figure 6A show that primer RAPD1 produced 17 different bands, primer RAPD 2 (Figure 6B) generated 16 different band patterns, and primer RAPD 3 produced 14 different band patterns (Figure 7A). Data in Figure 7B show the generated bands produced from primer RAPD4, which was produced for differentiation. The molecular weights of these bands produced from the four examined primers were between 150 bp and 950 bp. These bands were selected, cut, purified, and sequenced. Five specific genes (bands) from strain *B. thermophilus*, which were induced when treated with *Mentha spicata* oil, were selected for sequencing. The sequence analysis revealed that these genes corresponded to lipid kinase, extracellular solute-binding protein, naphthoate synthase, major facilitator superfamily, and transglycosylase (Table 6). Further search on the Internet regarding the function of each gene confirmed that they are all involved in the defense pathway mechanism.

**Table 6:** Sequence similarities of over-expressed or new DD fragments generated from Real-time PCR of *Bacillus thermophilus* cells treated with *Mentha spicata* oil compared with known genes from BLASTx searches in NCBI.

**DD band size bp**	**Biological function**	**Similar microorganism**	**Similarity %**
204	Lipid kinase	*Pseudomonas mendocina*	32%
618	Extracellular solute-binding protein	*Streptomyces himastatinicus*	48%
633	Naphthoate synthase	*Actinomyces coleocanis*	29%
553	Major facilitator super family	*Bacillus cereus*	67%
543	transglycosylase	*Lactobacillus*	28%

## 3. Discussion

Sugar beet is an important source of sugar production worldwide, contributing ~27% of total sugar production. The countries that produce a high amount of sugar beet are Japan, the United States, and Eastern Europe. In Egypt, it is the second main and biggest source of sugar production after sugar cane. The sugar beet industry often faces problems such as losing sucrose during the storage period or the production process. During the storage period, a large group of microorganisms infect the fruit bodies of sugar beet and cause the loss of sucrose by converting it to other sugars. Consequently, our targets were directed to know the most common microorganisms that attack fruits during storage and search for bio-antimicrobial substances from natural sources such as plant extracts (Shittu et al., 2007; Landete et al., 2008; Agatemor, 2009; Qadrie et al., 2009) or oils (Vahdati-Mashhadian and Rakhshandeh, 2005; Matos et al., 2006), which can be used to inhibit the growth of these infecting microorganisms, aiming to reduce the rate of loss. To achieve the main goals of this study, samples were collected regularly during storage time from the four big industrial sectors (Alexandria, Dakahlia, Kafr El-Sheikh, and Sharkia) that produce sugar from beet in Egypt.

Screening for the most common bacteria that attack sugar beet during storage or manufacturing was performed by both biochemical (MicroScan Panels) and molecular analysis (16S rRNA).

According to the results of biochemical assays, it was determined that *Bacillus subtilis, Leuconostoc mesenteroides, Pseudomonas fluorescens, Escherichia coli, Acinetobacter baumannii, Staphylococcus xylosus, Enterobacter amnigenus*, and *Aeromonas* were the common mesophilic bacteria found to be attacking sugar beet fruits during storage before washing samples. However, after the washing process, only five species from the previously mentioned were detected. In contrast, a thermotolerant species called *Bacillus thermophilus* was the dominant bacterium attacking the sugar beet at elevated temperatures of 55–60°C based on the results of the MicroScan Panels. Since this bacterial species was the most dangerous and caused a lot of sucrose loss, sequencing of the 16S rRNA gene was conducted to confirm its identification. Molecular identification (16S rRNA) confirmed the identification using MicroScan Panels. Similar results were obtained by Fredsgaard et al. who noted that *Bacillus thermophilus* was the common thermotolerant species present in the juice of sugar beet (Fredsgaard et al., 2017). Other reports by Strom confirmed the identification of *Bacillus thermophilus* as a thermotolerant bacteria from solid-waste compost (Strom, 1985).

There are a number of traditional methods, such as carbamates (McGinnis, 1971), glutaraldehyde (Eager et al., 1986), and formalin, that are followed and applied by different sugar companies to limit the reduction in the yield of sugar in both processes by reducing the growth of these contaminants. However, the use of these chemicals is always associated with health problems. Therefore, our second goal is to find another safe and friendly alternative antibacterial agent using plant extracts (Burt, 2004; Vági et al., 2004; Agatemor, 2009) or oils (Vahdati-Mashhadian and Rakhshandeh, 2005; Matos et al., 2006) to prevent microbial contamination. Several authors have used both plant extracts (Meena and Mariappan, 1993; Singh et al., 1994; El-Korashy, 1997) or oils (Ramesh et al., 2006; Hajlaoui et al., 2009) as natural bactericidal products. Consequently, a comprehensive screening was conducted to analyze 20 plant extracts and 13 oils to identify a promising source that exhibits a high ability to inhibit the growth of the common thermopilic bacterium (*Bacillus thermophilus*). This particular bacterium has been previously reported to cause significant losses in sugar yield. Among the 20 plant extracts and 13 oils examined in our study, 18 plant extracts and 11 oils were effective with varied abilities to inhibit the contaminants that attack beets during storage or manufacturing. Plant extracts of *Geranium gruinum, Datura stramonium*, and *Mentha spicata* and the oils of *Mentha spicata* and *Ocimum bacilicum* were the most promising natural sources to reduce the presence of *B. thermophilus* by forming a high inhibition zone around it, which reached 40 mm, 39 mm, and 35mm, as well as 50 mm and 45 mm, respectively. These results are in agreement with those obtained by Singh, who confirmed the ability of *Mentha spicata* extract to be used as an antifungal against *Fusarium oxysporum* (Singh et al., 1994), as well as El-Korashy (El-Korashy, 1997), who reported that the extract of *Mentha spicata* could inhibit the growth of *Rhizoctonia solani* and *Fusarium solani*. Velluti confirmed our findings, as they found that the oils of cinnamon could reduce the harmful effect of infecting maize with *Fusarium proliferatum* (Velluti et al., 2003). The useful use of *Mentha spicata* oil as a strong antibacterial against *Bacillus thermophilus* in this study with a maximum inhibition zone of 50 mm was confirmed by several authors who noted previously in different scientific articles that the oil of *Mentha spicata* proved to be used as an antibacterial and antifungal against a wide range of microorganisms (Meena and Mariappan, 1993; Gulluce et al., 2006; Jirovetz et al., 2006; Hajlaoui et al., 2009).

The plant extracts that proved their ability to be used as antibacterials against microorganisms contain different chemical components, such as phenolic acids and flavonoids (Fabri et al., 2009; Aragão et al., 2010; Chisté et al., 2010). Further, oils contain terpenoids and aromatic compounds (Parcha et al., 2003), which acquire plants' biological functions as bactericidal or fungicidal. Moreover, the two most important oils, *Mentha spicata* and *Ocimum basilicum*, which significantly reduced the growth of *Bacillus thermophilus* in the two stages of sugar manufacturing (storage and production), were subjected to analysis by GC/MS.

The results obtained from GC and MS analysis of the plant extract from *Mentha spicata* revealed that the major constituent present was isopulegon, which constituted 12.29% of the extract. These results are consistent with those obtained by Tayarani-Najaran *et al*. and Boukhebti *et al.*, who confirmed the presence of isopulegon and pulegone as major components in the oil derived from this plant (Boukhebti et al., 2011; Tayarani-Najaran et al., 2013). Generally, the active compounds found in plant extracts, such as flavonoids, exert their antibacterial and antifungal effects by inhibiting the DNA gyrase enzyme (Cushnie and Lamb, 2005). However, terpenoids present in oils may disrupt the electron transport chain, oxidative phosphorylation, or the ability to dissolve the cytoplasmic membrane due to their lipophilic properties (Parcha et al., 2003).

The fourth goal was using the RAPD technique to detect genetic changes or recombinant DNA that can be generated after treating thermophilic bacteria (*B. thermophilus*) with effective oils due to the simplicity and applicability of this technique (Williams et al., 1990; Istock et al., 2001). The results of the RAPD method indicated that a number of differentiated bands (monomorphic or polymorphic) appeared due to the treatment with effective oils with the four RAPD primers used. However, the number of bands varied depending on the type of primer. The results ensure that oils contributed to changes in the genetic structure and function of the treated bacterial cells due to their antibacterial effects.

The five highly induced genes were observed in growing strain *B. thermophilus* on nutrient agar medium after being treated with the oil of *Mentha spicata* by DD-PCR and were selected for sequencing as the fifth goal. Sequencing results indicated that these five genes were similar to lipid kinase, extracellular solute-binding protein, naphthoate synthase, the major facilitator superfamily (MFS), and transglycosylase. All of these genes work together in one or different pathways to increase the defense or resistance of bacterial cells against oil, which is used to suppress the growth of microbes. These results are supported by Cain, who reported that the lipid kinase gene functioned as bacteriocin to help the bacterial cell resist the toxins through phosphorylation (Cain et al., 1993), and Tam and Saier noted that the extracellular solute-binding proteins are essential to facilitate the transport of organic solutes under different kinds of stresses (Tam and Saier, 1993). The Transglycosylase enzyme works as an exo-coenzyme in different biological processes inside cells (Beachey and Keck, 1033). The MFS proteins are known to be present as a major constituent of different microorganisms' genomes, and they are usually involved in the cycles of sugar uptake (Griffith et al., 1992).

## 4. Conclusion

In the present study, we screened a set of plant extracts and oils for their antibacterial activity against thermophilic bacteria (*B. thermophilus*) attacking sugar beet fruits during storage or production. Our findings indicated that most of the evaluated extracts possess considerable antibacterial activity to reduce the growth of thermal-resistant bacteria. Among the evaluated extracts, two oils from *Mentha spicata* and *Ocimum bacilicum* exhibited the most potent activity to inhibit the growth of *Bacillus thermophilius*, as indicated by inhibition zones and their ability to reduce the amount of lactic acid by 47.7% and 26.5%, respectively, indicating low sugar degradation. The characterization of *Mentha spicata* and *Ocimum bacilicum* by GC-MS analysis indicated the presence of antibacterial substances such as Linalool and isopulegone. Future studies should explore the applicability of these extracts in industry and scientific research.

## 5. Materials and methods

### 5.1. Isolation of bacteria from sugar beet roots and juice

To count mesophilic bacteria, 1 mL of juice was spread on the surface of tryptone soya agar medium (Scotter et al., 2000), and then, plates were incubated for three days at 37°C. Growing colonies were picked up and used to inoculate the tryptone soy broth medium. Afterward, 1 mL of this inoculated broth medium was distributed on the surface of the blood agar medium to isolate G+ve bacteria (DIFCO Laboratories Dehydrated, 1984), while the G-ve bacteria were obtained by distributing 1 mL of this culture on the surface of the MacConkey agar medium. To count and isolate the thermophilic bacteria, adopting the procedure of Sahm and Washington (Sahm et al., 1991), 1 mL of the serial dilutions was spread on the surface of the nutrient agar medium, and plates were incubated at 55°C for 48 h. Both isolated mesophilic and thermophilic bacteria were examined microscopically, and the common isolates were purified and subjected to complete identification through biochemical tests and molecular analysis.

### 5.2. Identification of bacterial isolates

Mesophilic bacterial isolates were subjected to complete identification using the MicroScan Dried system in the Microbiology lab at Mansoura Children's Hospital University, which is designed to identify both Gram-negative and positive bacteria using fluorogenic substrates as a pH indicator for a bacterial enzymatic reaction. Conventional MicroScan Negative and positive Combo panels were inoculated with the bacterial isolates using the standard turbidity technique. The panels were incubated for 24 h at 35°C within the MicroScanWalkAway system. All procedures were performed according to the manufacturer's instructions (Abdel-Wahab et al., 2022). It is also used for measuring several biochemical tests, including carbohydrate fermentation tests such as glucose, xylose, mannitol, sucrose, galactose, rhamnose, maltose, raffinose, sorbitol, arabinose, inulin, mannose, and lactose, in addition to other specific tests such as Voges Proskauer (VP), nitrate reduction (NIT), indole test, urease test, hydrogen sulfide production test, citrate utilization, and oxidase test.

### 5.3. Morphological and cultural characteristics of thermophilic bacterial isolates

Isolates were tested using classical microbiological methods such as Gram stain, spore-forming using a phase contrast microscope (Sala et al., 1995), and motility as described by Priest (Priest et al., 1988), in addition to examining their biochemical reactions such as the ability to grow under anaerobic conditions, starch hydrolysis, catalase test, Voges–Proskauer test, casein hydrolysis, and nitrate reduction. To examine the ability of strains to grow anaerobically, isolates were inoculated on nutrient agar tubes containing the following ingredients per liter: 20 g of Trypticase, 5 g of NaCl, 10 g of glucose, 15 g of agar, 2 g of sodium thioglycolate, and 1 g of formaldehyde sulfoxylate). Additionally, 1 ml of nutrient broth culture was added to each tube. Then, the growth of the strains was evaluated by the naked eye after incubating the tubes for 4 days at 55°C. The ability of strains to hydrolyze starch was tested by growing them on nutrient agar plates containing 1% starch (pH 6.5) and incubating them at 55°C for 3 days. The appearance of a clear zone around the colonies upon the addition of Lugol's iodine indicated the presence of amylase activity and the ability of starch hydrolysis. Catalase reaction strains were subjected to tests to determine whether or not they could hydrolyze hydrogen peroxide by adding drops of 3% H_2_O_2_ on the surface of the bacterial colony, and positive strains that produced bubbles were recorded against those negative strains that could not produce bubbles.

The Voges–Proskauer (VP) test is used to detect the presence of acetoin in bacterial broth cultures. The test was conducted by adding alpha-naphthol and potassium hydroxide to the Voges-Proskauer broth, which had been previously inoculated with a bacterial strain. A cherry-red color indicates a positive result, while a yellow-brown color indicates a negative result (MacFaddin, 2000). Additionally, the ability of these bacteria to hydrolyze casein was demonstrated by the production of a clearing halo zone around their colonies after they were grown on skimmed milk agar plates. The composition of the agar plates included 1.0% skimmed milk, 0.2 % yeast extract, 0.01 % KH_2_PO_4_, 0.03 % K_2_HPO_4_, 0.5% NaCl, and 2% agar (pH 6.5). The plates were incubated at 55°C for 72 h. This hydrolysis ability was described by Fujio and Kume (Fujio and Kume, 1991). A bacterial colony that gives off a red color after cultivation in a broth medium containing 5g peptone, 3g beef extract, and 1g KNO_3_, supplemented with naphthylamine and B sulfanilic acid, indicates positive nitrate reduction.

### 5.4. Molecular tools for identifying thermophilic bacteria

Bacterial genomic DNA was obtained from a bacterial isolate that was previously identified as *B. thermophilius* using a biochemical assay using the QIAGEN Genomic DNA Purification Kit according to the manufacturing procedure. Then, the pure DNA was evaluated under UV light after electrophoresis on a 0.8% agarose gel that had been supplemented with ethidium bromide in TBE buffer. 1 μl of pure bacterial DNA from this strain was subjected to RAPD-PCR. The RAPD-PCR method employed was described by Heikal et al. (Heikal et al., 2008). Four different primers, namely A1 (5,-GGACTGGAGTsGTGATCGC-3), Ez351 (5,-AGGAGGTGATCCAACCGCA-3), Rapid2 (5,-GTTACGCTCC-3), and A7a10 (5,-GAAACGGGTGGTGATCGCAG-3), were used. The RAPD-PCR reaction was conducted in a total volume of 25 μl, comprising 10 x buffer, 2 μl of 25 mM MgCl2, 2 μl of 2.5 mMdNTPs, 1 μl of each 10 pmol primer, 0.2 μl of Taq DNA polymerase (5 unites/μl), and 1 μl of 50 ng DNA as the template. The PCR reaction was conducted using the following program cycles; cycle at 95°C for 5 min; then, 40 cycles were performed as follows: For 1 min at 95°C for denaturation, 1 min at 30°C for annealing, 1 min at 72°C for elongation, and 10 min at 72°C for a final extension. Reaction mixtures were held at 4°C. The generated fragments were assessed on a 2% agarose gel containing ethidium bromide after electrophoresis in TBE buffer for 1 h.

To amplify the gene coding for 16S rRNA to identify the strain, primers 350F (5,-AGGTGATCCAACCGCA-3) and 350R (5,-AATGGAGGAAGGTGGGGAT-3) corresponding to the polymorphic region and conserved gene sequence of bacterial 16S rRNA (El-Hanafy et al., 2007; Mohamed et al., 2021, 2022a,b) were used to amplify ~350bp. The amplified fragment of 350 bp was assessed on a 0.8% agarose gel plus ethidium bromide after running in TBE for 30 min. After evaluating the targeted fragment of 16S rRNA, the fragment was purified using a PCR clean-up column kit (Maxim Biotech, Inc., USA).

The targeted and purified fragment of 16S rRNA was sent with the forward primer that was previously used for amplification to the Macrogen Company (South Korea) for sequencing. Sequences obtained from 16Sr RNA by the previously mentioned company were compared with the available sequences in the Gene Bank database using BLASTN searches at the National Center for Biotechnology Information site (http://ncbi.nlm.nih.gov).

Multiple DNA sequence alignments were carried out using the Clustal w program version 1.82 (http://www.ebi.ac.uk/clustalw) (Thompson et al., 1997; Abdel-Wahab et al., 2022).

Bacterial cells were lyzed in 1 mL of GS1 buffer after adjusting the cell number at a rate of 10^6^–10^7^ in sterile water. Then, total RNA was extracted from the treated and non-treated thermophilic bacterial strains using the GStract™ RNA Isolation kit II (GuanidiumThiocyanate) according to the manufacture's instructions, after taking into account that all the disposable plastics used in this method should be RNase-free, decontaminated, and treated with 0.1% v/v diethyl pyrocarbonate (DEPC) in water.

### 5.5. Collection of root beet samples

Sixty samples of sugar beet roots (10 beet roots for each sample) were collected from different companies in Dakahlia, Alexandria, Kafr El-Sheikh, and Sharkia and stored in mild conditions at Dakahlia Sugar Company for 29 days. Leaf petioles were trimmed at the base, and the terminal buds were removed. Then, the levels of sugar, Na, K, α amino-N, best quality, and inverted sugar, as well as temperature, were determined every 2 days during the storage period. Isolation, collection, and identification of the most common bacteria from all samples were achieved.

### 5.6. Collection of beet juice samples

Juice samples were collected from the middle extraction tower of Dakahlia Sugar Company at different time intervals (4 h), transferred to the laboratory in sterilized bottles, and kept at 4°C until further analysis. Sugar percentage cossets, sugar losses, and sugar yield were estimated for every sample.

### 5.7. Determination of sucrose losses

The levels of each apparent sugar (Na, K, α amino-N, beet quality, inverted sugar, and temperature) were determined every 2 days during storage. Sucrose was determined with a polar meter (sucromate), sodium (Na), potassium (K), and α-amino nitrogen were measured using the cold digestion method, while inverted sugars were detected using the Benzamidine method (Edye and Clarke, 1998; Larrahondo et al., 2006). Invert sugar percent was used as an indicator for sucrose losses. The Benzamidine method was used to estimate the reducing carbohydrate; to determine the optimal conditions of the benzamidine reaction, the reaction time, temperature, medium pH, reagent concentration, and the fluorescence spectrum have been investigated.

### 5.8. Determination of the sucrose losses during the manufacturing process

During beet sugar manufacturing, there are two types of losses: determined and undetermined. Determined losses were measured in the factory laboratory, and undetermined losses were calculated. Samples from pulp, mud, and molasses every day (during the campaign) were collected, and then the sugar level was determined by routine daily work. The average was determined every 10 days (period), and the undetermined losses and total losses were calculated.

### 5.9. Determination of lactic acid

Juice samples were collected from the BMA (Braunschweigerische Maschinenbauanstalt AG) extraction tower at Dakahalia Sugar Company. Juice samples were collected from the diffuser and incubated at 55°C. The lactic acid and pH were measured and collected using the lactic acid analyzer and pH meter (052-50910080, model 910/8) at different times. The drop in pH and increased lactic acid indicated high bacterial activity and sucrose destruction.

### 5.10. Preparation of plant extracts

All cultivated plants were collected from the Botanical Gardens and Ornamental Plants Department, Horticulture Research Institute (HRI) Institute, Agricultural Research Center, Egypt, while wild plants were collected and identified in the Botany Department, Mansoura University, Egypt, by Prof. Ibrahim A. Mashaly (Professor of Flora and Plant Ecology), stored in the herbarium of the Plant Department, Mansoura University, Egypt, with their herbarium ID as shown in Supplementary Table S1.

The leaves of plants Labiatae (*Ocimum basilicum* L.; *Mentha spicata* L. & *Mentha longifolia* L.), Rutaceae (Citrus limon L.), Lauraceae (*Cinnamomum zeylanicum* L.), Umbelliferae (*Cuminum cyminum* L.), Myrtaceae (*Eugenia caryophyllus* L. & *Eucalyptus rostrata* Schlecht.), Geraniaceae (*Geranium gruinum* L.), Verbenaceae *(Lantana camara* L.), Gramineae (*Cymbopogon proximus* (hochst) staps), Solanaceae (*Datura stramonium* L. & *Nicotiana glauca* R.C. Graham), Asteraceae (*Silybum marianum* Gaertn L. & *Calendula officinalis* L.), Cannabaceae (*Humulus lupulus* L.), Anacardiaceae (*Schinus terebenthifolius* Radd), Apocynaceae (*Nerium oleander* L.), and Zingibiraceae (*Zingiber officinale* L.) were collected from cultivated fields to use them for excreting the active gradients to be used as antibacterial substances against bacteria that grow on sugar beet during storage or manufacturing. Plant materials were dried at room temperature for 15 days, ground using an electric mill to obtain a fine powder, and extracted by soaking in 1:1 (w/v) methanol and shaking for 3 days. The soaker was filtered through a specific filter under strong hand pressure, and the solvent was removed under vacuum at 60–65°C to produce a crude extract using a rotary evaporator (Alade and Irobi, 1993). The crude extract was preserved in the fridge for further analysis, dissolved in dimethyl sulfoxide DMSO (1mg/mL), and diluted extract solutions were used for different analyses (Taylor et al., 1996; El-Sherbieny et al., 2002).

### 5.11. Selection of a strong antibacterial product to inhibit the growth of *B. thermophilus*

After obtaining the diluted extracts from 20 medicinal plants that were dissolved in DMSO, which proved its neutral effect on the growth of tested microorganisms, as it did not make an inhibition zone for growing *B. thermophilus*, aliquots of these extracts with a concentration of 50 μg/ml were used as an antimicrobial product to reduce the growth of the tested microbe using the agar diffusion technique, as previously explained by Hili (Hili et al., 1997). To test each extract, 50 μl containing 50 μg/ml of active ingredients (in nutrient broth inoculated with microbes) was added to the well made by a cork borer on the surface of nutrient agar medium, and plates were incubated for 18h at 55°C (Burt, 2004; Holley and Patel, 2004). The inhibition of growth due to the addition of antibacterial products was recorded by eye compared to the control, and the diameters were measured. Similarly, 13 oils from Labiatae (*Origanum majorana* L.; *Ocimum bacilicum* L.; *Thymus vulgaris* L.&*Mentha spicata* L.), Myrtaceae (*Eucalyptus rostrata*s chlecht; *Eugenia caryophyllus* L.&*Cinnamomum zeylanicum* Blume), Alliaceae (*Allium sativum* L.), Oleaceae (*Olea europaea* L.), Umbelliferae (*Apium graveolens* L.), Umbelliferae (*Pimpinella schweinfurthii* Asch), Asteraceae (*Prunus dulcisvar* L.), and Rutaceae (*Citrus limon* L.) that purchased from local Egyptian Market were examined against the growth of the tested bacteria.

### 5.12. GC/MS analysis of the oil composition of *Mentha spicata* and *Ocimum basilicum*

The two effective oils of *Mentha spicata* and *Ocimum basilicum* against the growth of thermophilic bacteria were analyzed using Gas Chromatography and Mass Spectrometry (GC/MS) at the National Research Center, Dokki, Cairo, Egypt, using a Varian 3400 GC equipped with a DB-5 fused silica capillary column (30m X 0.25 mm; i.d. μm film thickness). The multi-step temperature program used for analysis was raised from 60°C (held for 3 min) to 260°C (held for 10 min) every 5°C min^−1^. Helium was the carrier gas used at a flow rate of 1 ml min^−1^, and the sample size was 1 μL (injector temperature was 250°C). A mass spectrometer version of the Varian-Finnigan SSQ7000 was used to determine each peak's mass with an ionization voltage of 70 eV. Scan time and mass range were 5 s and 40–400 m/z, respectively. Different oil peaks were nominated by matching their recorded mass spectra with those stored in the Wiley/NBS mass spectral library of the GC/MS data system and other published mass spectra (Adams, 2001).

### 5.13. Reverse transcription-polymerase chain reaction of mRNA

Reverse transcription (RT), or first-strand DNA, was obtained after converting the mRNA to complementary DNA (cDNA) in the presence of dNTPs and reverse transcriptase. The components were combined with a DNA primer in a reverse transcriptase buffer for 1 h at 42°C. The exponential amplification via reverse transcription was conducted using a polymerase chain reaction (a highly sensitive technique), where a very low copy number of RNA molecules can be detected. A reverse transcription reaction was performed using an oligodT primer (5′-TTTTTTT -TTTTTTTT-3′). Each 25 μl reaction mixture contained 2.5 μl (5x) buffers with MgCl2, 2.5 μl (2.5 mM) dNTPs, 1 μl (10 pmol) primer, 2.5 μl RNA (2 mg/ml), and 0.5 units of reverse transcriptase enzyme. The PCR reaction was conducted using PCR programs at 42°C for 1 hr. and 72°C for 10 min (enzyme killing), and the product was stored at 4°C until use.

### 5.14. Differential display PCR and sequencing of targeted fragments

Primers RAPD1 (5′-TGCCGAGCTG-3′), RAPD2 (5′-ATGCCCCTGT-3′), RAPD3 (5′-AGCCACCGAA-3′), and RAPD4 (5′-CCTTGACGCA-3′) were included to conduct differential display analysis to examine genetic changes that occur due to the antibacterial effect (obtained as a result of oil addition) against the common microorganism that attacks sugar beet during storage or manufacturing (*Bacillus thermophilus*). The reaction of DD-PCR was carried out in 25μl containing 2.5 μl 10x buffer with MgCl2, 2μl (2.5 mM) dNTPS, 1 μl of 10 pmol primer, 1.5μl cDNA, and 0.2 μl (5 units/μl) Taq DNA polymerase. PCR is programmed as follows: 1 cycle at 95°C for 5 min, then 40 cycles for 30 sec at 95°C, 1 min at 30°C for annealing, and 1min at 72°C for elongation. The reaction was incubated at 72°C for 10 min for a final extension before being stored at 4°C until further use. Afterward, 10 μl of the differential PCR products were mixed with 2 μl of loading dye and electrophoresed at 80 volts in 0.5 x TBE running buffer. The gel was stained with 0.5 μg/cm3 (w/v) ethidium bromide and checked under UV light. Separated DNA bands of DD-PCR representing upregulated and downregulated genes were cut from the gel, purified using a gel extraction kit from Promega, USA, and sent with the primer to the same company mentioned before for sequencing.

## Data availability statement

The original contributions presented in the study are included in the article/Supplementary material, further inquiries can be directed to the corresponding authors.

## Author contributions

Conceptualization and writing—original draft preparation: MY, A-NZ, AD, EH, and ES. Validation: MY, A-NZ, SK, AA, RB, and MJ. Investigation: AD, AS, AA, HA, RB, MJ, and EH. Resources: MY, A-NZ, AA, RB, MJ, and EH. Data curation: MY, AD, AS, RB, HA, EH, and ES. Writing—review and editing: MY, A-NZ, AD, AS, SK, AA, RB, MJ, HA, EH, and ES. Visualization: MY, AD, HA, EH, and ES. Supervision: EH. Project administration and funding acquisition: HA, RB, and MJ. Methodology: All authors. All authors read and agreed to the published version of the manuscript.
