# Supplementary material for: Exploring the antibacterial potential of plant extracts and essential oils against Bacillus thermophilus in beet sugar for enhanced sucrose retention: a comparative assessment and implications
Source: Front Microbiol. 2023 Jul 20;14:1219823. doi: 10.3389/fmicb.2023.1219823 (PMC10400092; doi:10.3389/fmicb.2023.1219823)
Supplement: Supplementary file 1 [file Table_1.pdf]

Exploring the Antibacterial Potential of Plant Extracts and Essential Oils Against Bacillus thermophiles in Beet Sugar for Enhanced Sucrose Retention: Comparative Assessment and Implications

Mohamed M. Yousef [1], Abdel-Naser A. Zohri [2], Amira M. G. Darwish [3,4*], Abdelaal Shamseldin [5], Sanaa A. Kabeil [6], Ahmed Abdelkhalek [7], Reem Binsuwaidan [8], Mariusz Jaremko [9], Hussah Abdullah Alshwyeh [10,11], Elsayed E. Hafez [7], Essa M. Saied [12,13,*]

Table S1. Identification of wild plants with herbarium ID used in this study.

Plant name	Family	ID
Eucalyptus rostrataschlecht	Myrtaceae	MansH 520
Geranium gruinum L.	Geraniaceae	MansH 233
Lantana camara L.	Verbenaceae	MansH670
Cymbopogonproximus (hochst) staps	Gramineae	MansH 270
Daturastramonium L.	Solanaceae	MansH 788
Nicotianaglauca R.C. Graham	Solanaceae	MansH 797
SilybummarianumGaertn L.	Asteraceae	MansH 189
Calendula officinalis L.	Asteraceae	MansH 167
Humuluslupulus L.	Cannabaceae	MansH 207
SchinusterebenthifoliusRadd	Anacardiaceae	MansH 177
Nerium oleander L.	Apocynaceae	MansH 193
